# Susceptibility of White-Tailed Deer to Rift Valley Fever Virus

**DOI:** 10.3201/eid2409.180265

**Published:** 2018-09

**Authors:** William C. Wilson, In Joong Kim, Jessie D. Trujillo, Sun Young Sunwoo, Leela E. Noronha, Kinga Urbaniak, D. Scott McVey, Barbara S. Drolet, Igor Morozov, Bonto Faburay, Erin E. Schirtzinger, Tammy Koopman, Sabarish V. Indran, Velmurugan Balaraman, Juergen A. Richt

**Affiliations:** US Department of Agriculture, Manhattan, Kansas, USA (W.C. Wilson, L.E. Noronha, D.S. McVey, B.S. Drolet, E.E. Schirtzinger);; Kansas State University College of Veterinary Medicine, Manhattan (I.J. Kim, J.D. Trujillo, S.Y. Sunwoo, K. Urbaniak, I. Morozov, B. Faburay, T. Koopman, S.V. Indran, V. Balaraman, J.A. Richt)

**Keywords:** white-tailed deer, Odocoileus virginianus, Rift Valley fever virus, RVFV, viruses, arbovirus, susceptibility, epidemiology, livestock, zoonoses, United States

## Abstract

Rift Valley fever virus, a zoonotic arbovirus, poses major health threats to livestock and humans if introduced into the United States. White-tailed deer, which are abundant throughout the country, might be sentinel animals for arboviruses. We determined the susceptibility of these deer to this virus and provide evidence for a potentially major epidemiologic role.

Rift Valley fever virus (RVFV) is a zoonotic, arthropodborne RNA virus (order *Bunyavirales*, family *Phenuiviridae*, genus *Phlebovirus*) (*1*,*2*). The virus is maintained in nature in a mosquito–vertebrate host cycle and is endemic to sub-Saharan Africa where epidemics have great consequences for livestock and human health. There is potential for RVFV incursions into neighboring regions or introductions into other continents, including North America, which has mosquito species capable of harboring and transmitting RVFV (*3*).

Although domestic cattle, sheep, and goats are susceptible to RVFV and function as amplification hosts during epidemics, the potential role of wildlife host species, such as white-tailed deer (*Odocoileus virginianus*) is unknown. RVFV is capable of infecting a range of cell lines from wildlife in North America, including white-tailed deer (*4*), suggesting in vivo susceptibility. White-tailed deer might be good sentinel animals for various arboviruses because of their abundance and wide geographic distribution in the United States (*5*).

A serious concern is that white-tailed deer could serve as reservoir or amplification hosts for RVFV (*6*–*9*). Furthermore, modeling suggests that these deer as reservoir hosts would enhance spillover of RVFV into human populations because of overlap of mosquitoes, humans, and wildlife in urban and periurban areas (*7*). Therefore, we determined the susceptibility of white-tailed deer to RVFV infection and described the potential role of white-tailed deer populations in RVFV epidemiology.

## The Study

Wild-type RVFV (Kenya 2006 strain 128B-15, KEN06) was propagated in C6/36 mosquito cells and prepared as inoculum (1 × 10^6^ PFU/animal). Five 5-month-old male white-tailed deer from the US Department of Agriculture, Agricultural Research Service, National Animal Disease Center (Ames, IA, USA) captive herd were acclimated to Biosafety Level 3 conditions and housed in such a facility with specifically designed paneling.

After sedation and blood collection (0 days postinoculation [dpi]), 4 animals were injected subcutaneously in the neck with virus inoculum; 1 contact control animal was sham inoculated with cell culture medium. To minimize stress, 2 animals in the virus-inoculated group were sedated on alternating days (2–6 dpi) and then at 10 and 14 dpi for blood collection and physical examination. The control was sedated and sampled at 2, 4, 6, and 7 dpi. Animals were initially monitored 2 times/day, then 3 times/day after development of fever.

We determined blood levels of albumin, alkaline phosphatase, γ-glutamyl transferase, aspartate aminotransferase, and blood urea nitrogen by using a VetScan VS2 Analyzer (Abaxis, Union City, CA, USA). We used a quantitative reverse transcription PCR (qRT-PCR) to detect RVFV RNA (*10*). We performed humane euthanasia and necropsy when deer were moribund or at the end of the study (14 dpi). All animal work was performed at the Biosecurity Research Institute, Kansas State University (Manhattan, KS, USA), in compliance with Institutional Animal Care and Use Committee protocol no. 3518.

The 5 deer adapted well to the specifically designed room. Rectal temperatures were in the standard range (37.5°C–40.1°C) (*11*) at 0 dpi (Table). On dpi 2, clinical assessment of 2 infected deer (nos. 43 and 44) and the control (no. 41) showed that 1 inoculated animal (no. 44; 41.3°C) and the control (41.2°C) had increased body temperatures. Also, the control was highly agitated during capture.

**Table:** Assessment of Rift Valley fever virus infection in 5 white-tailed deer at selected days postinfection*

Group, animal no.	Day postinoculation, real-time qRT-PCR/virus isolation results, temperature, °C							
	0	2	3	4	6	7	10	14
Mock								
41	−/−, 39.2	−/−, **41.0**	NC	+/+, **39.6**	+++/+++, 40.7	+++/+++, **41.2**,** e**uthanized	NA	NA
Ken 06								
43	−/−, 39.3	++/++, **40.2**	NC	+/+, 39.0	–/–, **40.6**	NC	−/−, 39.1	−/−, 39.0
44	−/−, 39.4	+++/+++, **41.3**	+++/+++, died	NA	NA	NA	NA	NA
47	−/−, 39.5	NC	+++/+++, died	NA	NA	NA	NA	NA
52	−/−, 39.2	NC	NC	+/+, 39.3	–/–, 39.9	NC	−/−, 39.5	−/− , 39.1

*Bold indicates increased body temperature. NA, not available; NC, not collected; qRT-PCR, quantitative reverse transcription PCR; –, negative; +, cycle threshold range 31–35, 1 × 10^1^–1 × 10^3^ PFU/mL; ++, cycle threshold range 25–30, 1 × 10^4^–1 × 10^6^ PFU/mL; +++, cycle threshold <25, 1 × 10^7^–1 × 10^8^ PFU/mL.

qRT-PCR analysis of RNA isolated from serum samples obtained 2 dpi showed high concentrations of circulating virus RNA in deer no. 44 (8.15 × 10^10^ copies/mL) and high concentrations in deer no. 43 (3.0 × 10^7^ copies/mL). We did not detect RVFV RNA in serum from the control at 2 dpi. Later that day, 2 deer (nos. 44 and 47) were less active, and diffuse bilateral hyperemia of the ocular sclera developed in deer no. 44. At 3 dpi, bloody diarrhea developed in these 2 deer, and they died suddenly. qRT-PCR showed high serum levels of RVFV RNA (1 × 10^11^ copies/mL in deer no. 44 and 1 × 10^12^ copies/mL in deer no. 47).

Necropsy findings were similar for both animals and included severe, multifocal, hemorrhagic hepatic necrosis; moderate to severe segmental to diffuse hemorrhagic enteritis; moderate pulmonary edema; and moderate to severe hemorrhagic lymphadenopathy (Figure). Hepatic necrosis and petechiae have also been found in cattle and sheep with acute RVFV infections (*12*,*13*). Enteric lesions appeared to be severe and unique to white-tailed deer. Bloody fecal material covered the perineum, ventral tail, and hind limbs. Segmental hemorrhage of gastrointestinal mucosa was most severe in deer no. 44. We found watery and bloody gastrointestinal contents from the abomasum to the rectum (deer no. 44) or small intestine to the rectum (deer no. 47). Mesenteric and gastrohepatic lymph nodes of both animals were edematous and had multifocal hemorrhagic foci. We observed diffuse thymic hemorrhage in deer no. 44.

**Figure:**
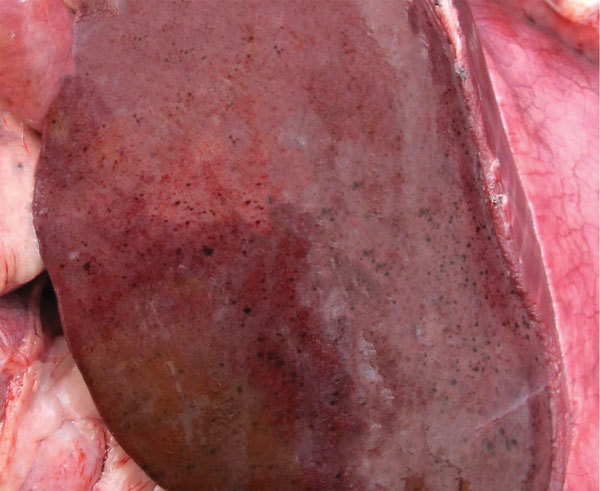
Gross pathologic view of liver of white-tailed deer no. 44 after experimental infection with Rift Valley fever virus inoculum. The animal died at day 3 postinoculation; at necropsy, the liver showed severe, multifocal, hemorrhagic hepatic necrosis attributed to acute infection with Rift Valley fever virus.

The remaining animals were bright, alert, and responsive at 3 dpi. However, 2 deer (nos. 43 and 52) had transient diarrhea with loose feces persisting until 6 dpi. qRT-PCR of serum showed moderate levels of RVFV RNA in deer no. 43 and deer no. 52. We also detected a low concentration (1 × 10^3^ copies/mL) of RVFV RNA in the control by 4 dpi, indicative of horizontal transmission. On day 6, the control and 1 inoculated deer (no. 43) had slightly increased body temperatures (40.7°C for the control and 40.6° for no. 43). By day 6, serum RVFV RNA concentration for the control had increased to ≈1 × 10^10^ copies/mL, and concentrations of virus RNA in deer no. 47 and no. 52 had decreased to 1 × 10^2^−1 × 10^3^ copies/mL. By 7 dpi, the control was recumbent and febrile (41.2°C) and marked swelling of the left hind limb had developed, which warranted euthanasia.

At necropsy for the control, hepatic and gastrointestinal lesions attributed to RVFV infection were similar to those in deer no. 44 and no. 47, albeit much less severe. Examination of the markedly swollen left hind limb showed marked expansion of subcutis and fascia with hemorrhage and emphysema but definite diagnosis is pending further investigation. RVFV infection of the control was supported by the high serum level of RVFV RNA at 7 dpi (8.1 × 10^8^ copies/mL). Serum qRT-PCR showed RVFV RNA in deer no. 43 (1 × 10^3^ copies/mL) but not in deer no. 52 at 10 dpi. By 14 dpi (end of the study), we did not detect RVFV RNA in serum of either remaining animal. We did not observe gross lesions in the remaining deer (nos. 43 and 52) at the end of the study. However, we detected RVFV RNA in liver, kidneys, spleen, and lymph nodes from both animals.

## Conclusions

Clinical signs, gross pathology, and qRT-PCR–determined virus RNA loads demonstrated that white-tailed deer are highly susceptible to RVFV infection, causing hepatic necrosis and hemorrhage. Supporting this conclusion, we found that levels of aspartate aminotransferase increased in serum of all animals when blood was collected at the time of clinical illness (range 91–153 U/L at 0 dpi and 629–3,543 U/L at the time of clinical illness). Similar results were reported for previous experimental RVFV infections of domestic cattle and sheep (*12*,*13*).

In addition, and unique to this study, RVFV infection in white-tailed deer resulted in development of hemorrhagic enteritis and bloody diarrhea at the time of peak viremia in 2 infected deer (nos. 44 and 47), which likely enabled horizontal transmission of RVFV to the control animal. Additional laboratory analysis is ongoing. However, our results clearly indicate that white-tailed deer in North America are susceptible to RVFV infection. Infected white-tailed deer died from the infection (n = 2), might survive the infection (n = 2), and can transmit the virus through direct contact (n = 1) presumptively by the fecal–oral route.

This study indicates that white-tailed deer in North America are highly susceptible to RVFV and capable of horizontal virus transmission. The potential role of other wildlife in the epidemiology of RVFV should be evaluated.
